# Development, evaluation and comparison of machine learning algorithms for predicting in-hospital patient charges for congestive heart failure exacerbations, chronic obstructive pulmonary disease exacerbations and diabetic ketoacidosis

**DOI:** 10.1186/s13040-024-00387-9

**Published:** 2024-09-12

**Authors:** Monique Arnold, Lathan Liou, Mary Regina Boland

**Affiliations:** 1 Department of Emergency Medicine, The Mount Sinai Hospital at the Icahn School of Medicine, 306 E 96th Street, #4A, New York, NY 10128 USA; 2 Icahn School of Medicine at Mount Sinai Hospital, New York City, NY USA; 3 Data Science, Department of Mathematics, Herbert W. Boyer School of Natural Sciences, Mathematics, and Computing, Saint Vincent College, Latrobe, PA USA

**Keywords:** Machine learning, Health informatics, Clinical informatics, Algorithms, Healthcare costs

## Abstract

**Background:**

Hospitalizations for exacerbations of congestive heart failure (CHF), chronic obstructive pulmonary disease (COPD) and diabetic ketoacidosis (DKA) are costly in the United States. The purpose of this study was to predict in-hospital charges for each condition using machine learning (ML) models.

**Results:**

We conducted a retrospective cohort study on national discharge records of hospitalized adult patients from January 1st, 2016, to December 31st, 2019. We constructed six ML models (linear regression, ridge regression, support vector machine, random forest, gradient boosting and extreme gradient boosting) to predict total in-hospital cost for admission for each condition. Our models had good predictive performance, with testing R-squared values of 0.701-0.750 (mean of 0.713) for CHF; 0.694-0.724 (mean 0.709) for COPD; and 0.615-0.729 (mean 0.694) for DKA. We identified important key features driving costs, including patient age, length of stay, number of procedures, and elective/nonelective admission.

**Conclusions:**

ML methods may be used to accurately predict costs and identify drivers of high cost for COPD exacerbations, CHF exacerbations and DKA. Overall, our findings may inform future studies that seek to decrease the underlying high patient costs for these conditions.

**Supplementary Information:**

The online version contains supplementary material available at 10.1186/s13040-024-00387-9.

## Background

### Healthcare costs associated with our outcomes: CHF, COPD, DKA

In the United States, hospital expenditures account for approximately one-third of overall healthcare expenditures, with an estimated total of US$1.192 billion in 2019 according to the Center for Medicare & Medicaid Services [1]. Healthcare costs are disproportionately concentrated among a small group of high-cost patients [2–4]. High-cost patients often have significant unmet critical healthcare needs despite the substantial healthcare costs they incur [5, 6].

Congestive heart failure (CHF), chronic obstructive pulmonary disease (COPD) and diabetes mellitus are life-altering, high-cost, high-volume conditions that affect millions of people and result in many hospitalizations per year [7]. According to Medical Expenditure Panel Survey data for 2017 to 2018 published by the American Heart Association (AHA), diabetes mellitus, heart disease, CHF and respiratory conditions, including COPD, were among the top 10 leading diagnoses for direct health expenditures [8].

CHF is one of the leading causes of hospitalization in the U.S., affecting 6 million adults as of 2018 and costing the nation an estimated $30.7 billion in 2012 according to the American Heart Association, with these costs deriving largely from exacerbations requiring emergency visits and hospitalizations [8–10]. Similarly, COPD is a high-cost disease–as COPD progresses, patients often experience acute exacerbations, characterized by dyspnea, cough, sputum production and worsening lung function; COPD exacerbations cause frequent hospital admissions and readmissions, reportedly accounting for 90.3% of the total medical cost related to COPD and leading to US $32.1 billion in total medical cost [11, 12]. Finally, diabetic ketoacidosis (DKA) is one of the acute, life-threatening complications of diabetes mellitus, a disease affecting 37.3 million people as of 2019 according to the CDC [13]. DKA is a common cause of hospitalization in patients with diabetes and is characterized by uncontrolled hyperglycemia, metabolic acidosis, and increased serum ketone concentrations [14, 15].

### Prior machine learning methods studying our outcomes: CHF, COPD, DKA

Machine learning (ML) techniques have emerged as a mechanism for analyzing high-dimensional medical data to understand the factors underlying patient-, hospital- and health system-level outcomes [16]. Specifically, for our three cohorts of patients, ML techniques have been utilized to identify at-risk patients, predict the risk of readmission and readmission rates, and predict the length of inpatient stay [11, 12, 17–21]. Work has been done to develop predictive models to identify major underlying drivers of high healthcare costs for patients in generalized cohorts as well as several other cohorts of patients, such as breast cancer patients and coronary artery bypass graft patients [22–26]. To date, however, robust machine learning algorithms for predicting in-hospital expenditures and the factors that influence them have not been evaluated in patients experiencing CHF exacerbations, COPD exacerbations or DKA.

## Methods

The purpose of our study was to build and evaluate ML models to predict in-hospital charges associated with hospitalizations for these conditions, as this has not been done previously. Furthermore, based on the model output, we provide recommendations for model optimality in modeling in-hospital expenditures in each cohort and identify factors that underlie high-cost in-hospital admissions for each of the three diseases.

**Fig. 1:**
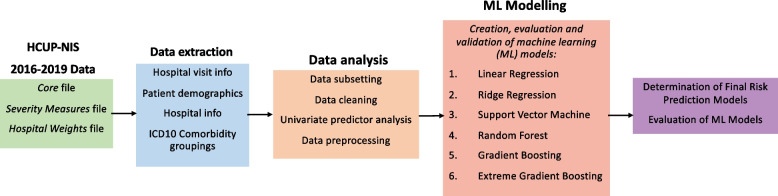
Overview of Study Methodolog: The HCUP-NIS 2016 Core, Severity Measures, Hospital Weights, and Cost Charge files were merged, and data related to hospital discharge and demographics were extracted as continuous, categorical and binary variables. ICD-10 comorbidity mappings from AHRQ were determined from ICD-10 codes. R codes were written to extract, clean and analyze the HCUP-NIS data. Six ML models were then trained, evaluated, and validated for each of the three disease cohorts, and the best model for each disease cohort was determined

An overview of the methodology employed is shown in Fig. 1. All data processing and statistical and machine learning analyses were conducted on a MacBook Air (2022) equipped with an Apple M2 chip, 8 GB of unified memory, running macOS Sonoma (version 14.4.1). To optimize computational efficiency, we implemented parallel processing in R (version "Kick Things", released August 8, 2021) using the RStudio (version 1.4.1717) integrated development environment. We implemented models with *tidymodels, ranger, xgboost, glmnet* and *kernlab* packages of R.

### Dataset and Study Design

The National (Nationwide) Inpatient Sample (NIS) is a large, publicly available all-payer inpatient care database in the United States that contains data on more than seven million hospital discharges each year and is maintained as part of the Healthcare Cost and Utilization Project (HCUP) [27–29]. We used the HCUP-NIS Core, Severity, Hospital and Cost Charge datasets and queried the datasets for all hospitalizations between January 1, 2016, and December 31, 2019. Patients who were discharged from the hospital, patients aged < 18 years or who died were excluded.

We identified patients who met the three disease conditions using the International Classification of Diseases version 10 (ICD-10) codes: 1) chronic obstructive pulmonary disease (COPD) exacerbation via the ICD-10 code J441; 2) congestive heart failure (CHF) exacerbation via the ICD-10 codes I5021, I5023, I5031, I5033, I5041, and I5043; and 3) diabetic ketoacidosis without coma (DKA) via the ICD-10 codes E1010, E1011, E1111, and E1110 [30]. Supplemental Table 1 shows the extracted ICD-10 codes and principal diagnoses for each of these conditions.

We identified a total of 26,190 unique discharges across the three conditions, including 9,552 discharges for COPD, 14,688 for CHF and 1,950 discharges for DKA. The primary outcome for this study was total in-hospital charges.

### Predictor Variables

We conducted a preliminary literature review to determine potential factors that may affect in-hospital charges and that could be used as predictors in our analysis. The initial predictors for analysis included 46 variables, including 29 unique ICD-10 diagnosis code groupings extracted from the HCUP-NIS dataset, which included demographic characteristics, hospital-related variables, health care utilization six months before index admission, and discharge-related variables. A brief description of each predictor variable is given in Supplemental Table 2. Further descriptions of the potential values of each variable can be found on the NIS Description of Data Elements page (https://www.hcup-us.ahrq.gov/db/nation/nis/nisdde.jsp).

The ICD10 diagnosis codes were transformed into Agency for Healthcare Research and Quality (AHRQ) comorbidity categories using the *icd* R package. If a patient had at least one ICD10 code in one of the AHRQ comorbidity categories, then they were considered positive for that category. A list of AHRQ comorbidity categories is shown in Supplemental Table 3.

### Univariate Analysis of Predictor Variables

The relationships between each of the predictor variables and total charges were analyzed using two-sample t tests. Statistical significance was determined at the 95% confidence level, with *p* < 0.05 indicating statistical significance. We also calculated the correlations between each predictor variable in the dataset using the Pearson method. To reduce the quantity of variables without having to choose variables a priori*,* only variables with a Pearson correlation coefficient above 0.2 were visualized.

### Model Specification

We investigated six ML algorithms: linear regression (LM), ridge regression (Ridge), support vector machine (SVM), random forest (RF), gradient boosting (GBM) and extreme gradient boosting (XGB). These are popular models used in machine learning for healthcare classification and prediction. First, we preprocessed the variables using common feature engineering steps as described in “Preprocessing and Feature Engineering of Predictor Variables” section. Then, we split the data for each condition into training and testing datasets, with 75% of the derivation sample for in-sample training and 25% for out-of-sample testing. Next, we performed hyperparameter tuning for our six algorithms using a randomized grid search and 5-fold cross-validation and determined the best hyperparameters as described in “Hyperparameter Tuning” section. The final model with tuned hyperparameters for each algorithm was then fit to the testing data using 5-fold cross-validation as described in “Model Finalization” section. We then evaluated the performance of each model as described in “Model Performance Evaluation and Comparison” section, then examined the final feature importance rankings as described in “Assessment of Feature Importance” section.

#### Preprocessing and Feature Engineering of Predictor Variables

Due to the asymmetric distribution of characteristics and predictor variables, cases with missing data for any of the dependent or independent variables were excluded from this analysis, a common, though controversial, approach for dealing with missing values [31]. Then “one-hot encoding” was performed, transforming each categorical variable into a numerical dummy variable, a common preprocessing step to aid analyses with different ML models [32]. Next, within the dataset for each condition, variables with zero variance and those with large absolute correlations with other variables were determined and excluded from the datasets [33]. Finally, all continuous or numerical predictor variables were standardized such that their mean was 0 and standard deviation was 1 (Z-score standardization). This is a common preprocessing method used to decrease the likelihood of bias of the model due to very large or small numeric variables [34]. After this preprocessing, the preprocessed datasets for each condition contained the 46 preprocessed predictor variables.

#### Hyperparameter Tuning

Where applicable, we performed a randomized grid search for hyperparameter tuning to optimize model performance, generalizability and robustness on unseen data [33]. An overview of the considered hyperparameters is displayed in Supplemental Table 4. Hyperparameter ranges were chosen based on those used in prior work [33, 35, 36]. Model performance for each hyperparameter permutation was assessed using fivefold cross-validation to determine the optimal settings that achieved the best balance between bias and variance. The top-performing model was determined as that hyperparameter permutation for each model that produced the best R-squared when fitted to the out-of-sample test dataset.

#### Model Finalization

The tuning hyperparameter combinations with the best mean R-squared values across fivefold cross validation was used in the final model for each algorithm for each condition. These final models were then fit to the training dataset, then used to predict total charges based on the testing dataset.

#### Model Performance Evaluation and Comparison

Performances of the final models were estimated by their R-squared and root-mean square error (RMSE), which are common metrics used to measure the accuracy of prediction models [37, 38]. R-squared is a measure of the goodness of fit of a model and has a maximum value of 1. Models with R-squared values closer to 1 are more well fitted to the data. RMSE measures the quality of predictions by determining how far predictions fall from measured true values using the Euclidean distance. It is a standard metric for measuring the error of a model, with smaller values indicating less random noise and thus higher accuracy. Model performance according to these two metrics on the in-sample training set and out-of-sample testing set were determined. Top-performing models were determined best on R-squared estimates.

#### Assessment of Feature Importance

Importance of the predictors in the final models were determined from their variable importance (VI) scores. VI scores demonstrate how much the prediction changes as the feature values vary, with higher feature importance indicates greater importance of the feature to the model prediction [39]. For linear models, the relative importance is determined by the absolute value of the t-statistic. For gradient boosting models, the relative importance is determined from the absolute value of the coefficients corresponding to the tuned model. Based on this relative feature importance, we visualized the top twenty most influential features in VI plots (VIPs).

## Results

### Sample characteristics

**Fig. 2:**
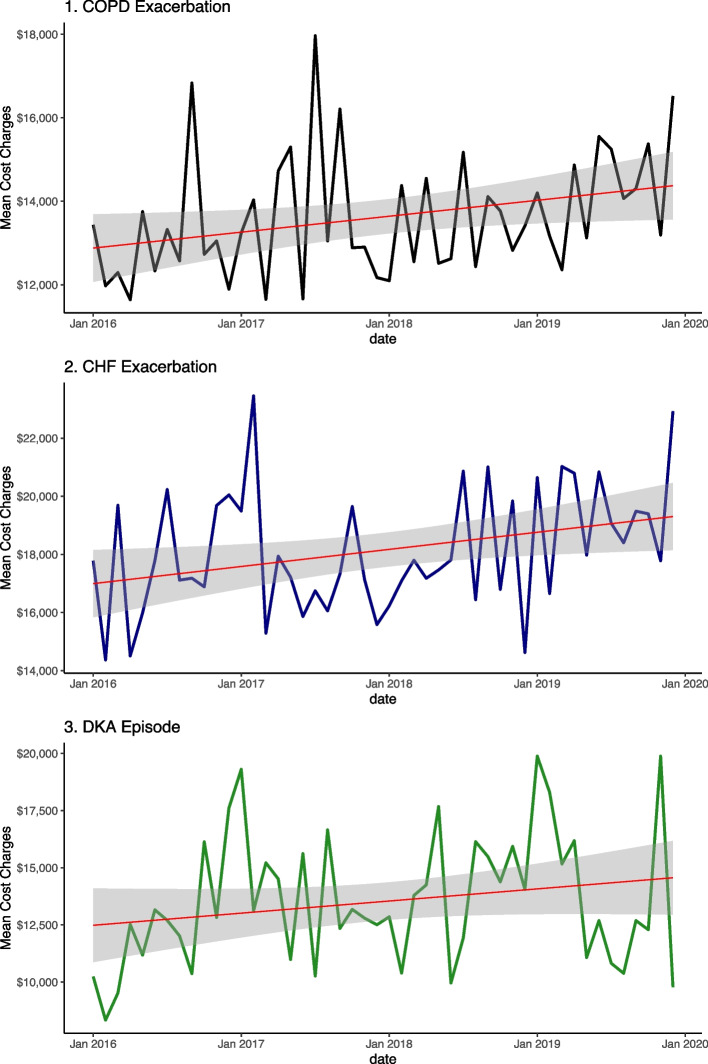
Mean Cost Charges: Trends in mean cost charges for hospitalization for each condition, 2016-2019

In total, 26,190 unique hospital discharge records with complete data were available for the analysis from January 1, 2016, to December 31, 2019–14,688 patients hospitalized for CHF exacerbation, 9,552 patients hospitalized for COPD exacerbation and 1,950 patients hospitalized for DKA without coma. The characteristics of the sample cohorts are summarized in Table 1. The average costs for hospitalizations were US$18,196 (± $29,248) for CHF exacerbations, US$13,572 (± $17,598) for COPD exacerbations and $13,650 (± $16,778) for DKA episodes. The mean length of stay and number of inpatient procedures were highest in the CHF cohort at 6.36 days and 1.90 procedures, respectively; the mean length of stay was 5.32 days in the COPD exacerbation cohort and 5.08 days in the DKA cohort, and the number of procedures was 1.32 for both COPD patients and DKA patients. As shown in Fig. 2**,** the mean cost charges for each condition steadily increased for each condition over the four-year period from 2016 to 2019.

**Table 1:** Patient sample characteristics

	**CHF Exacerbation**	**COPD Exacerbation**	**DKA Episode**	**Overall**
**n**	14688	9552	1950	26190
**Total Charges ($, mean (SD))**	18196.21 (29247.63)	13572.25 (17598.13)	13650.48 (16777.65)	16171.31 (24876.85)
**Length of Stay in days (mean (SD))**	6.36 (7.26)	5.32 (5.74)	5.08 (6.75)	5.89 (6.73)
**Number of Procedures (mean (SD))**	1.90 (2.82)	1.32 (2.13)	1.32 (2.31)	1.64 (2.57)
**Elective Admission = Yes (%)**	1910 (13.0)	1155 (12.1)	194 (9.9)	3259 (12.4)
**Sex = Female (%)**	7521 (51.2)	5385 (56.4)	1029 (52.8)	13935 (53.2)
**Age (mean (SD))**	66.77 (17.84)	64.92 (16.51)	50.11 (19.78)	64.85 (18.03)
**Race (%)**				
White	10500 (71.5)	7243 (75.8)	1208 (61.9)	18951 (72.4)
Black	2166 (14.7)	1165 (12.2)	402 (20.6)	3733 (14.3)
Hispanic	1197 (8.1)	663 (6.9)	214 (11.0)	2074 (7.9)
Asian Pacific Islander	374 (2.5)	195 (2.0)	54 (2.8)	623 (2.4)
Native American	73 (0.5)	56 (0.6)	23 (1.2)	152 (0.6)
Other	378 (2.6)	230 (2.4)	49 (2.5)	657 (2.5)
**Insurance status (%)**				
Medicare	9621 (65.5)	6086 (63.7)	707 (36.3)	16414 (62.7)
Medicaid	1827 (12.4)	1345 (14.1)	504 (25.8)	3676 (14.0)
PrivateInsurance	2570 (17.5)	1647 (17.2)	514 (26.4)	4731 (18.1)
SelfPay	375 (2.6)	268 (2.8)	162 (8.3)	805 (3.1)
NoCharge	23 (0.2)	22 (0.2)	10 (0.5)	55 (0.2)
Other	272 (1.9)	184 (1.9)	53 (2.7)	509 (1.9)
**Median household income quartile for patient ZIP Code (%)**				
0 to 25th percentile	4017 (27.3)	2877 (30.1)	635 (32.6)	7529 (28.7)
26th to 50th percentile	3771 (25.7)	2613 (27.4)	498 (25.5)	6882 (26.3)
51st to75th percentile	3767 (25.6)	2314 (24.2)	464 (23.8)	6545 (25.0)
76th to 100th percentile	3133 (21.3)	1748 (18.3)	353 (18.1)	5234 (20.0)
**Discharge (%)**				
Routine	7633 (52.0)	5545 (58.1)	1393 (71.4)	14571 (55.6)
Transfer to Hospital	321 (2.2)	175 (1.8)	38 (1.9)	534 (2.0)
Transfer to Other Facility	3578 (24.4)	1917 (20.1)	278 (14.3)	5773 (22.0)
Home Health Care	3155 (21.5)	1914 (20.0)	241 (12.4)	5310 (20.3)
Unknown	1 (0.0)	1 (0.0)	0 (0.0)	2 (0.0)
**Patient Location (%)**				
"Central" counties of metro areas of > = 1 million population	4421 (30.1)	2712 (28.4)	626 (32.1)	7759 (29.6)
"Fringe" counties of metro areas of > = 1 million population	3688 (25.1)	2404 (25.2)	449 (23.0)	6541 (25.0)
Counties in metro areas of 250,000-999,999 population	2953 (20.1)	1876 (19.6)	408 (20.9)	5237 (20.0)
Counties in metro areas of 50,000-249,999 population	1361 (9.3)	981 (10.3)	195 (10.0)	2537 (9.7)
Micropolitan counties	1160 (7.9)	811 (8.5)	141 (7.2)	2112 (8.1)
Not metropolitan or micropolitan counties	1105 (7.5)	768 (8.0)	131 (6.7)	2004 (7.7)
**Hospital Division (%)**				
New England	1190 (8.1)	546 (5.7)	92 (4.7)	1828 (7.0)
Middle Atlantic	3094 (21.1)	2100 (22.0)	368 (18.9)	5562 (21.2)
East North Central	866 (5.9)	547 (5.7)	79 (4.1)	1492 (5.7)
West North Central	3581 (24.4)	2480 (26.0)	493 (25.3)	6554 (25.0)
South Atlantic	1370 (9.3)	871 (9.1)	166 (8.5)	2407 (9.2)
East South Central	1240 (8.4)	942 (9.9)	165 (8.5)	2347 (9.0)
West South Central	716 (4.9)	375 (3.9)	130 (6.7)	1221 (4.7)
Mountain	522 (3.6)	377 (3.9)	102 (5.2)	1001 (3.8)
Pacific	2109 (14.4)	1314 (13.8)	355 (18.2)	3778 (14.4)
**Hospital Bedsize (%)**				
Small	2044 (13.9)	1621 (17.0)	278 (14.3)	3943 (15.1)
Medium	3497 (23.8)	2526 (26.4)	467 (23.9)	6490 (24.8)
Large	9147 (62.3)	5405 (56.6)	1205 (61.8)	15757 (60.2)
**Hospital Location/Teaching Status (%)**				
Rural	916 (6.2)	808 (8.5)	126 (6.5)	1850 (7.1)
Urban Non-Teaching	2431 (16.6)	1931 (20.2)	323 (16.6)	4685 (17.9)
Urban Teaching	11341 (77.2)	6813 (71.3)	1501 (77.0)	19655 (75.0)
**Hospital Control/Ownership (%)**				
Government, nonfederal	1653 (11.3)	1101 (11.5)	295 (15.1)	3049 (11.6)
Private, not-profit	11710 (79.7)	7396 (77.4)	1464 (75.1)	20570 (78.5)
Private, invest-own	1325 (9.0)	1055 (11.0)	191 (9.8)	2571 (9.8)

### Univariate analyses

Tables 2 and 3 show the univariable results for the categorical and continuous variables, respectively. A longer inpatient stay and greater number of procedures were associated with greater in-hospital total charges. Older patients also incurred higher total charges. For several features, such as sex, payment method, hospital bedsize, hospital control, hospital location, All Patients Refined Diagnosis Related Groups (APRDRG) severity score and APRDRG risk mortality score, the differences in total charges between groups of patients within each cohort were often statistically significant (for example, patients in large hospitals incurred greater charges than those in smaller hospitals in each disease cohort, *p* < 0.05). Notably, black patients incurred more charges than white patients did (*p* < 0.01).

**Table 2:** Univariable results for categorical variables

	**CHF**			**COPD**			**DKA**		
	**Mean Charges**	***p*-value**		**Mean Charges**	***p*-value**		**Mean Charges**	***p*-value**	
**Sex**									
Male	$20,362			$14,909			$14,095		
Female	$16,132	0.00	****	$12,538	0.00	****	$13,253	0.27	ns
**Race**									
White	$18,067			$13,319			$13,926		
Black	$17,379	0.60	ns	$13,645	0.84	ns	$10,917	0.01	**
Hispanic	$18,139	0.94	ns	$13,812	0.84	ns	$15,967	0.48	ns
Asian Pacific Islander	$21,378	0.24	ns	$17,079	0.16	ns	$14,833	0.84	ns
Native American	$18,276	0.94	ns	$14,366	0.84	ns	$18,703	0.60	ns
Other	$23,489	0.16	ns	$17,326	0.16	ns	$15,487	0.84	ns
**Payment Method**									
Private Insurance	$19,193			$13,285			$12,951		
Medicare	$18,070	0.35	ns	$13,758	0.48	ns	$16,398	0.00	**
Medicaid	$17,609	0.31	ns	$13,264	0.98	ns	$12,071	0.48	ns
Self-Pay	$15,916	0.11	ns	$11,673	0.35	ns	$9,106	0.00	**
No Charge	$9,612	0.00	**	$11,102	0.48	ns	$5,410	0.00	**
Other	$21,041	0.42	ns	$15,327	0.21	ns	$14,246	0.64	ns
**Hospital Bedsize**									
Small	$13,775			$12,446			$12,447		
Medium	$15,270	0.00	**	$12,862	0.41	ns	$11,750	0.51	ns
Large	$20,303	0.00	****	$14,242	0.00	****	$14,665	0.04	*
**Hospital Location**									
Rural	$11,894			$11,007			$10,736		
Urban Non-Teaching	$13,773	0.00	**	$11,706	0.13	ns	$11,896	0.30	ns
Urban Teaching	$19,653	0.00	****	$14,405	0.00	****	$14,273	0.00	***
**Hospital Control**									
Government	$18,839			$14,660			$14,077		
Private Not Profit	$18,593	0.87	ns	$13,649	0.29	ns	$13,905	0.87	ns
Private Inves tOwn	$13,887	0.00	****	$11,896	0.00	**	$11,043	0.06	ns
**APRDRG Severity**									
Minor LOF	$16,181			$11,425			$10,375		
Moderate LOF	$16,092	0.88	ns	$11,796	0.38	ns	$11,615	0.15	ns
Major LOF	$18,092	0.00	**	$14,015	0.00	****	$14,894	0.00	****
Extreme LOF	$29,323	0.00	****	$23,751	0.00	****	$26,027	0.00	****
No Class	$21,596	0.65	ns	$10,116	0.80	ns			
**APRDRG Risk Mortality**									
Minor Likelihood of Dying	$16,550			$11,534			$10,724		
Moderate Likelihood of Dying	$16,349	0.72	ns	$12,733	0.00	***	$13,435	0.00	**
Major Likelilhood of Dying	$18,513	0.00	***	$14,786	0.00	****	$16,753	0.00	****
Extreme Likelihood of Dying	$28,668	0.00	****	$23,321	0.00	****	$23,799	0.00	****
No Class	$21,596	0.68	ns	$10,116	0.72	ns			

* indicates statistical significance at the 5% level (p < 0.05), ** indicates statistical significance at the 1%level (p < 0.01), *** indicates statistical significance at the 0.01% level (p < 0.001), and **** indicates statistical significance at the 0.001% level (p < 0.0001)

**Table 3:** Univariable results for continuous variables

	**CHF**				**COPD**				**DKA**			
	**Estimate**	**Std. Error**	**t value**	**Pr ( >|t|)**	**Estimate**	**Std. Error**	**t value**	**Pr ( >|t|)**	**Estimate**	**Std. Error**	**t value**	**Pr ( >|t|)**
Age	-1.38	10.90	4.16	0.00	45.36	10.90	4.16	0.00	173.85	18.81	9.24	0.00
Length of Stay	3024.61	21.94	137.86	0.00	2344.85	20.21	116.02	0.00	1694.71	41.16	41.17	0.00
Number of Procedures	6399.13	67.30	95.09	0.00	4880.51	68.06	71.71	0.00	4556.15	128.55	35.44	0.00

**Fig. 3:**
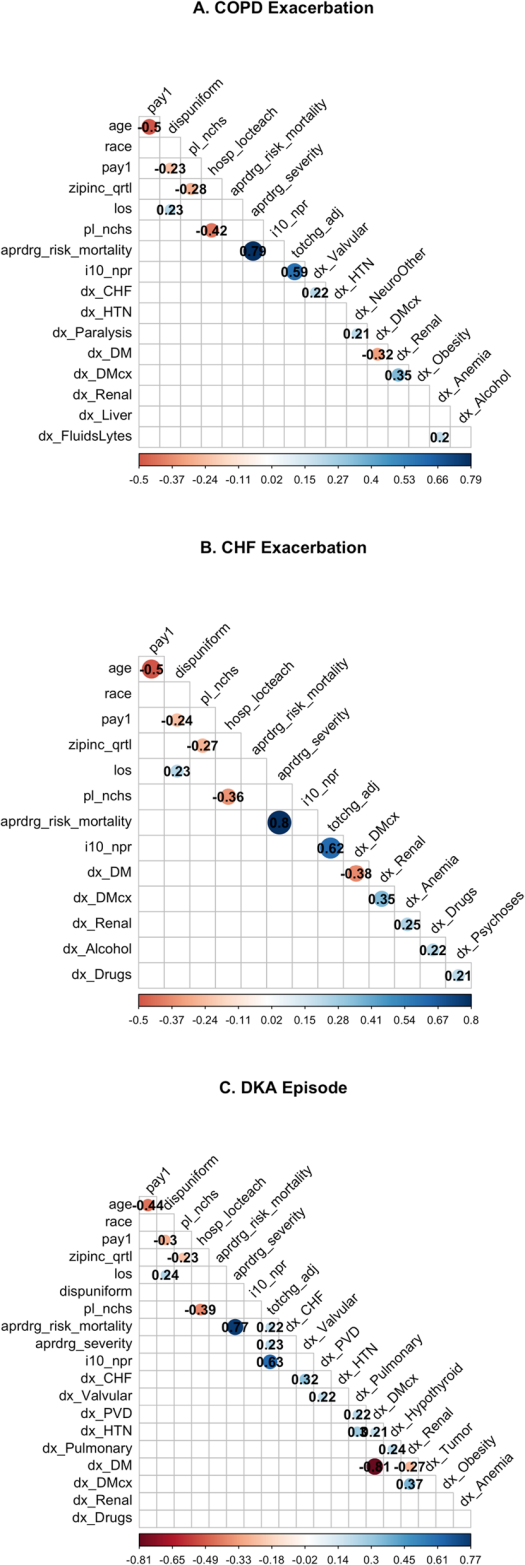
Correlation plots with Pearson coefficients for variables each disease condition dataset. Only those variables with a Pearson coefficient > 0.2 are displayed

The Pearson coefficients of the most correlated variables are visualized in Fig. 3. The data show that collinearity exists between several variables. For each of the three conditions, the number of procedures and APRDRG risk mortality were the most strongly positively correlated with the nondiagnosis variables (with correlation coefficients of 0.80 for CHF, 0.79 for COPD and 0.77 for DKA), while age and payment method were the most negatively correlated with the nondiagnosis variables (with correlation coefficients of -0.50 for CHF, -0.50 for COPD and -0.44 for DKA).

### Model results

**Fig. 4:**
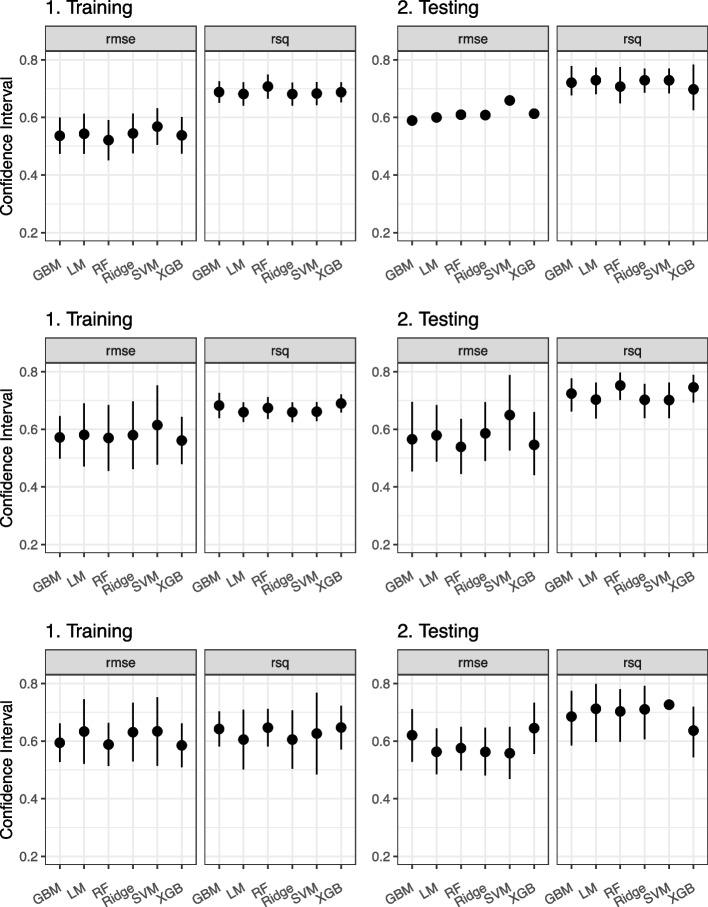
Comparison of Evaluation Metrics: Comparison of the RMSE and R-squared values and their confidence intervals for each final model each condition

Table 4 and Fig. 4 show the comparison of the RMSE and R-squared values and their confidence intervals for training and testing each model applied to each medical diagnosis.

**Table 4:** Comparison of the evaluation metrics of the ML models

	**CHF**				**COPD**				**DKA**			
	**Train**		**Test**		**Train**		**Test**		**Train**		**Test**	
**Model**	**RMSE**	***R***^2^	**RMSE**	***R***^2^	**RMSE**	***R***^2^	**RMSE**	***R***^2^	**RMSE**	***R***^2^	**RMSE**	***R***^2^
LM	0.581	0.659	0.580	0.703	0.543	0.681	0.603	0.724	0.633	0.605	0.563	0.717
Ridge	0.580	0.659	0.585	0.701	0.544	0.681	0.616	0.722	0.631	0.605	0.565	0.715
SVM	0.614	0.661	0.647	0.701	0.568	0.683	0.670	0.724	0.634	0.625	0.560	0.729
RF	0.568	0.676	0.542	0.750	0.519	0.710	0.622	0.694	0.589	0.646	0.575	0.706
GBM	0.586	0.671	0.591	0.693	0.536	0.688	0.601	0.714	0.583	0.651	0.620	0.679
XGB	0.566	0.681	0.558	0.730	0.545	0.680	0.631	0.677	0.608	0.634	0.687	0.615
**Mean**	**0.582**	**0.668**	**0.584**	**0.713**	**0.542**	**0.687**	**0.624**	**0.709**	**0.613**	**0.628**	**0.595**	**0.694**

Supplemental Fig. 1 show the top 20 features in each final model for each condition as determined by their V1 scores. Length of stay had the highest V1 scores in all models for all conditions, indicating it was the most important predictor in each of the models. The number of procedures during hospitalization was consistently the second most important feature, with age and elective/nonelective admission also consistently being strong predictors across the models. This finding aligns with our univariable analyses (Tables 2 and 3).

## Discussion

Although many studies have employed ML techniques to predict at-risk patients, readmission risks, readmission rates and length of stay for CHF, COPD and DKA patients, the development of a predictive model of in-hospital cost charges in these disease cohorts is a novel contribution of this study.

We constructed 6 ML models that had good predictive performance. The R-squared values ranged from 0.659 to 0.681 with a mean of 0.668 during training and 0.701 to 0.750 with a mean of 0.713 during testing for the CHF dataset; from 0.680 to 0.710 with a mean of 0.687 during training dataset and 0.694 to 0.724 with a mean of 0.709 during testing for COPD; and from 0.605 to 0.651 with a mean of 0.628 for training and 0.615 to 0.729 with a mean 0.694 during testing for DKA. As such, on average, models similarly for all three conditions and, on average, models performed better on the unseen testing data than on the training data.

Unsurprisingly, length of stay was the most important predictor in each of the models, disproportionately affecting hospital charges in each model. This was followed by the number of procedures performed during hospitalization. Age and elective/nonelective admission were also important predictors in at least one model for each disease condition. Feature selection indicates that although these variables are extremely influential in any model, many other patient-level and hospital-level features also have small but measurable impacts on hospital charges.

### Strengths of our study

The strengths of our study include the large sample size of the HCUP NIS datasets. Furthermore, the availability of many demographic characteristics, diagnosis-related variables, and hospital characteristics for use as predictors allowed for the building of supervised prediction models. The use of advanced ML techniques represents the robust use of data science to characterize complex clinical issues. The ability to predict expenditures at the patient level with good accuracy can allow for targeted care by anticipating the health care needs of patients. This will provide insights into designing effective and tailored interventions to meet the needs of high-cost patients and reduce costs.

### Limitations of our study

Despite its strengths, we recognize that this work has several limitations. Missing data are a well-known limitation of utilizing EMR data for research, for which the HCUP-NIS is susceptible. Additionally, we chose to use only complete data without missing values for all predictor variables, thereby eliminating a substantial number of possible discharge events. Future work can involve employing data imputation methods rather than data exclusion. This could help to address the potential selection bias that can result from categorically excluding cases with missing data.

Additionally, the discharge data used may include discharge from readmissions of the same patient. The NIS data contain discharge-level records, which, per the HCUP-NIS documentation, means that “individual patients who are hospitalized multiple times in one year may be present in the NIS multiple times… this will be especially important to remember for certain conditions for which patients may be hospitalized multiple times in a single year” [29, 40]. As discussed, our target patients often experience numerous hospitalizations, and initial versus recurrent hospitalizations might differ in their character. As such, we considered limiting the analysis to initial discharge; however, “…there is no uniform patient identifier available that allows a patient-level analysis with the NIS.” Therefore, for the purposes of this study, we included all the discharge data and performed the analysis at the discharge level.

## Conclusion

We demonstrated the use of ML models to predict in-hospital charges for patients hospitalized for CHF exacerbation, COPD exacerbation and DKA. We found that length of stay, number of procedures during hospitalization, age and elective/nonelective admission were important predictors in these models for these diseases. This research can provide helpful information for medical management, which may decrease health insurance burdens in the future.

## Supplementary Information

Supplementary Material 1.

## Data Availability

The data that support the findings of this study are available from Agency for Healthcare Research and Quality but restrictions apply to the availability of these data, which were used under license for the current study, and so are not publicly available. Data are however available from the authors upon reasonable request and with permission of Agency for Healthcare Research and Quality.

## Data availability

No datasets were generated or analysed during the current study.
