# Supplementary material for: Development, evaluation and comparison of machine learning algorithms for predicting in-hospital patient charges for congestive heart failure exacerbations, chronic obstructive pulmonary disease exacerbations and diabetic ketoacidosis
Source: BioData Min. 2024 Sep 12;17:35. doi: 10.1186/s13040-024-00387-9 (PMC11395859; doi:10.1186/s13040-024-00387-9)
Supplement: Supplementary file 1 — Supplementary Material 1. [file 13040_2024_387_MOESM1_ESM.docx]

**SUPPLEMENTAL FIGURES AND TABLES**

**Supplemental Table 1.** Conditions of interest and their associated ICD-10 diagnosis codes. Diagnoses were mapped from the ICD-10-CM/PCS MS-DRG v37.0 Definitions Manual.

**Supplemental Table 2.** Predictor/independent variables employed in the ML analysis.

**Supplemental Table 3.** Comorbidity predictor variables mapped from ICD-10 codes and their AHRQ CSS definitions.

**Supplemental Table 4.** Grid search space of the hyperparameters of the algorithm models. The columns min and max indicate the regions from which samples of these hyperparameters are drawn. *p* refers to the number of variables in the dataset. Linear regressions typically do not involve hyperparameters for tuning; consequently, the LM model was fitted using its default settings without any tuning, and is not listed above. We used the *grid_space_filling* function from the *dials* R package in the *tidymodels* ecosystem using Latin hypercube sampling to generate a space-filling design of hyperparameter combinations, enabling efficient exploration of the parameter space during model tuning. For each model 20 possible permutations were tested. The optimal hyperparameter value from tuning was determined from the grid permutation that produced the best R-squared for each model.

**1A.** **COPD Exacerbation**

**1B.** **CHF Exacerbation**

**1C.** **DKA Episode**

**Supplemental Figure 1.** VIPs for top-performing model for algorithm for each disease condition as determined from the final models.
